# Subchronic Exposure to Arsenic and Nickel Triggers Nitrosative Stress and Histological Alterations in the Rat Liver

**DOI:** 10.1002/jat.70146

**Published:** 2026-03-12

**Authors:** Thainá Iasbik‐Lima, Luiz Otávio Guimarães‐Ervilha, Isabela Pereira da Silva Bento, Renê Chagas da Silva, Mariana Machado‐Neves

**Affiliations:** ^1^ Departamento de Biologia Geral Universidade Federal de Viçosa Viçosa Minas Gerais Brazil; ^2^ Departamento de Física Universidade Federal de Viçosa Viçosa Minas Gerais Brazil

**Keywords:** hepatotoxicity, histomorphometry, inflammation, metal mixture, nitric oxide

## Abstract

Environmental exposure to heavy metals poses a threat to human health. Several studies documented harmful effects on the liver after arsenic and nickel exposure at concentrations beyond permissible limits. Nevertheless, the impact of a simultaneous exposure to both elements on hepatic biochemistry and histology remains unelucidated. Therefore, this study aimed to evaluate the effects of individual and combined ingestion of arsenic and nickel on the rat liver. Adult males (*n* = 10/group) received saline (control), 1 mg L^−1^ of arsenic, 7 mg L^−1^ of nickel, and two metals simultaneously in drinking water for 70 days. The results showed that arsenic at 1 mg L^−1^ did not alter ionic and oxidative parameters (*p* > 0.05), whereas nickel at 7 mg L^−1^ increased the proportion of Ca and Cu, the occurrence of protein oxidation, and the Ca^2+^ ATPase activity (*p* < 0.05). Under our experimental conditions, no alterations were observed in hepatic MDA levels or in serum ALT, AST, and ALP (*p* > 0.05). Histological alterations in the rat liver after isolated arsenic and nickel exposure, including vascular congestion, hydropic degeneration, and inflammatory infiltrate, were intensified after metal co‐exposure. The vascular disorder affected the proportion of hepatic components (blood vessels, sinusoids, cytoplasm, and hepatocytes; *p* < 0.05) in co‐exposed rats. Nickel increased the number of mast cells in exposed and co‐exposed rats (*p* < 0.05). Our findings revealed that subchronic co‐exposure to arsenic and nickel triggered nitrosative stress in the liver, culminating in histological alterations involving inflammation and vascular disorders mainly.

## Abstract 2

The investigation of co‐exposure to metals is of great toxicological and environmental importance. This study evaluated the hepatic effects after subchronic ingestion of As (1 mg L^−1^), Ni (7 mg L^−1^), and As+Ni in rats for 70 days. In contrast to individual exposure, co‐exposure intensified hepatic damage through ionic imbalance, oxidative damage, and ATPase dysfunction. It reduced the iron proportion and CAT activity, increased NO levels and mast cell count, and intensified the severity of vascular congestion and inflammatory infiltration.

## 1 Introduction

In recent decades, the increasing demand for exploiting Earth's natural resources has exacerbated environmental pollution (Chowdhury et al. 2018; Briffa et al. 2020). The bioavailability of chemical elements, including heavy metals, has become a global concern due to their persistent, nonbiodegradable nature and toxic potential to the environment and living organisms (Rehman et al. 2018; Fu and Xi 2020). Heavy metals are chemical elements with high atomic weights and densities (> 5 g cm^−3^), including metallic elements (e.g., nickel) and metalloids (e.g., arsenic), which are highly toxic and capable of causing harmful effects on human health even at low concentrations (Jomova et al. 2011; Briffa et al. 2020). Arsenic and nickel are real examples of chemical elements that pose combined risks to ecosystems and human health, as they coexist in both natural geological settings and contaminated areas (Vandeuren et al. 2023).

Several studies have reported that arsenic and nickel damage cells, disorganize tissue, and provoke physiological disorders (Pari and Prasath 2008; Adedara et al. 2019; da Cunha Meiros et al. 2019). This metalloid is often associated with changes in the kidneys, heart, liver, testis, and brain (Chiou et al. 2008; Noman et al. 2015; Souza et al. 2020). Nickel, in turn, may cause neurobehavioral and organic dysfunctions (Das and Buchner 2007; Pari and Prasath 2008; Adedara et al. 2020; Renu et al. 2021; Guo et al. 2023). Individual or combined exposure to arsenic and nickel can occur in various ways, as they are found in the air, soil, and water (Nielsen et al. 1984; Orloff et al. 2009; Khan et al. 2019). Humans can be exposed to both elements through consumer products, such as tobacco used in cigarette manufacturing and medicinal plants (Li et al. 2019; Sulaiman et al. 2024; Sandal et al. 2025), and occupational activities, often resulting from industrial processes that involve refining, smelting, and burning fossil fuels (Tokar et al. 2012). Overall, nickel is found in concentrations three to five times higher than arsenic (Vandeuren et al. 2023; Sulaiman et al. 2024; Sandal et al. 2025). The most relevant exposure route is through ingesting contaminated water and food at concentrations higher than the tolerated levels (0.07 mg L^−1^ of nickel; 0.01 mg L^−1^ of arsenic) established by governmental organizations (WHO 2011; CETESB 2014).

Recently, the field of environmental epidemiology has shifted to studying environmental exposures in ways that more closely resemble real‐life exposure mixtures (Martinez‐ Morata et al. 2022; Yin et al. 2024). Indeed, epidemiological studies revealed the importance of metal mixtures affecting populations worldwide and causing carcinogenic and metabolic issues (Chen et al. 2015; Li et al. 2019; Vormittag et al. 2021). Thus, new approaches are needed to assess the risks of simultaneous exposure to environmental toxicants. For that reason, researchers have dedicated efforts to understand the cause‐and‐effect of metal mixture poisoning (Adedara et al. 2017; Owumi et al. 2020; Arbi et al. 2021; Cao et al. 2024). Metal interactions can influence their absorption, resulting in additive, synergistic, antagonistic, or independent effects on general toxicity in exposed organisms (Driessnack et al. 2017). Such studies are relevant and timely, as there is limited information on the toxicities and associated mechanisms of heavy metal mixtures (Wu et al. 2016).

Notwithstanding, the liver can serve as a model of metal toxicity due to its role in detoxifying and synthesizing several substances for body homeostasis, including cholesterol, bile, and enzymes (Gu and Manautou 2012). Although several studies have documented the toxic effects of arsenic and nickel on liver parameters in adult animals (Fatoki and Badmus 2022; Apiamu et al. 2023; Sadighara et al. 2023), it is unclear whether simultaneous exposure to these elements can damage the liver. Therefore, we aimed to evaluate the effect of individual and combined exposure to arsenic and nickel on the liver of adult Wistar rats. We hypothesize that the co‐exposure to these toxic agents intensifies the tissue damage in the rat liver. To that end, we focused on hepatic metal accumulation and its consequences on histological, biochemical, functional, and oxidative parameters.

## 2 Materials and Methods

### 2.1 Animals and Ethics Statement

This study is part of a comprehensive work about the combined impact of subchronic exposure to arsenic and nickel on the liver and male reproductive organs of Wistar rats (Lima 2022). Seventy‐day‐old male Wistar rats (*N* = 40) were provided by the Central Animal Facility of the Universidade Federal de Viçosa (UFV). The animals were housed individually in polypropylene cages under controlled temperature (21°C) and photoperiod (12:12‐h light/dark cycles). All rats had access to rat chow (Nuvilab) and drinking water. The latter was used for treatment (oral route). The study was evaluated and approved by the Ethics Committee for the Use of Animals of the UFV (Protocol 45/2021). It was conducted in accordance with the ethical guidelines of the National Council for the Control of Animal Experimentation (CONCEA).

### 2.2 Experimental Design

Adult rats were randomly divided into four experimental groups (*n* = 10/group). Control animals received 0.9% saline solution, whereas the other exposed groups comprised animals ingesting 1 mg L^−1^ of arsenic (As group), in the form of sodium arsenite (AsNaO_2_; Sigma‐Aldrich Co., St. Louis, MO, USA), and 7 mg L^−1^ of nickel (Ni group), in the form of nickel chloride (NiCl_2_.6H_2_O; Sigma‐Aldrich Co., St. Louis, MO, USA). The co‐exposed group (As+Ni group) had rats receiving a solution containing 1 and 7 mg L^−1^ of arsenic and nickel simultaneously. Arsenite and nickel chloride are chemical forms highly soluble in water (Chen et al. 2005; Machado‐Neves and Souza 2023). The solutions were provided in filtered water daily for 70 days (subchronic exposure; Tokar et al. 2012). Metal concentration, in turn, was determined using 100 times the maximum allowable concentration of each metal, considering its presence in contaminated areas (Heikkinen et al. 2002; Rahman et al. 2009; WHO 2011) and differences in potency and toxicokinetics of each metal (Tokar et al. 2012; Owumi et al. 2020; Arbi et al. 2021; Cao et al. 2024). Water consumption was monitored daily to calculate the ingestion of metal compounds. In the end, rats from the As and Ni groups consumed, respectively, 0.12 ± 0.01 mg kg^−1^ of arsenic and 0.78 ± 0.05 mg kg^−1^ of nickel daily. Animals exposed to both elements consumed 0.11 ± 0.01 mg kg^−1^ of arsenic and 0.79 ± 0.06 mg kg^−1^ of nickel. We did not observe any alteration in animal clinical signs (weight loss, diarrhea, hair loss, vomiting, skin lesions, and nosebleeds) during the experimental period.

### 2.3 Euthanasia, Tissue Collection, and Biometric Analysis

After 70 days of the experiment, the animals were weighed and euthanized by deep anesthesia (xylazine 30 mg kg^−1^ i.p. and ketamine 300 mg kg^−1^ i.p.) followed by cardiac puncture. Blood was used to analyze serum hepatic enzymes using biochemical kits. The liver was removed and weighed to obtain the absolute weight and liver somatic index, which was calculated by normalizing the liver weight by the final body weight × 100 (Gonçalves et al. 2012). The liver was fragmented and immediately frozen in liquid nitrogen and stored at −80°C for oxidative/nitrosative stress and ATPase assays. Other fragments were fixed and used for histological analysis. Water content (mL g^−1^) was also measured using fresh hepatic tissue. For this, the tissue was dried at 60°C for 96 h and weighed to obtain its dry weight. Then, the difference between the wet and dry liver weight represents the water content (mL g^−1^; Novaes et al. 2012).

### 2.4 Functional Markers of Hepatic Damage

Blood samples (*n* = 6/group) were centrifuged at 2000 × g for 15 min at room temperature. Serum samples were used to assess the levels of alanine aminotransferase (ALT), aspartate aminotransferase (AST), and alkaline phosphatase (ALP) for evaluating hepatocellular integrity. *ALT and AST are direct and sensitive indicators of cytoplasmic leakage and hepatocyte membrane damage, whereas ALP is a widely used marker of liver injury in rodent toxicological studies* (Souza et al. 2018). The analysis was performed using biochemical kits (Bioclin Laboratories, Belo Horizonte, MG, Brazil) according to the manufacturer's instructions.

### 2.5 Histological Processing, Histopathological, and Stereological Analyses

Liver fragments (*n* = 6/group) were dehydrated in increasing ethanol series (70%, 80%, 90%, 95%, absolute), cleared in xylol, and embedded in paraffin. Sections with a thickness of 5 μm were obtained using a rotary microtome (RM 2255, Leica, Nussloch, Germany) and stained with hematoxylin and eosin (HE) for histopathological and stereological analyses, Periodic acid–Schiff (PAS) staining for glycogen, Masson's Trichrome for connective tissue, Sirius red for collagen, and toluidine blue for mast cells. Sections were evaluated in a semiseries (using 1 in every 10 sections) to avoid repetitive analysis of the same histological area.

Sections of liver tissue stained with HE were evaluated using a microscope (Olympus CX40, Tokyo, Japan) with 400× magnification. The presence and frequency of histological alterations, such as vascular congestion (evident accumulation of erythrocytes in the vascular lumen), hemorrhage (presence of extravasated erythrocytes in the extravascular space), inflammatory infiltrate (presence and accumulation of inflammatory cells in the hepatic parenchyma), and hydropic degeneration (increase in the volume of hepatocytes, with pale and vacuolated cytoplasm, resulting from the accumulation of intracellular fluid), were categorized as discrete (one histological field and one animal per group), moderate (3 histological fields and 2–3 animals per group), and severe occurrence (observed in ≥ 4 histological fields and ≥ 4 rats per group; Carvalho et al. 2022).

Ten histological images (200× magnification) per animal, stained in PAS, Masson's Trichrome, Sirius red, and toluidine blue, were obtained using an optical microscope (Olympus BX‐53, Tokyo, Japan) connected to a digital camera (Olympus DP73, Tokyo, Japan). They were analyzed by the following parameters using ImageJ software (National Institutes of Health). A grid system with 266 points was projected onto HE‐stained sections to perform stereological analysis. We recorded coincident points over components of liver parenchyma, including cytoplasm and nuclei of hepatocytes, sinusoidal capillaries, blood vessels, and macrophages (Kupffer cells). The proportion of each tissue component was calculated using the ratio of the number of points located in the structure of interest to the total number of points in the histological area (Mandarim‐de‐Lacerda 2003). Glycogen and connective tissue proportions (%) were obtained by counting pixels in sections stained with PAS and Masson's Trichrome. The collagen proportion (%), in turn, was recorded by measuring pixels in liver sections stained with Sirius Red and analyzed under an optical microscope with polarized light. Finally, the quantification of mast cells in the liver was performed using 10 histological fields stained with toluidine blue. They were counted in each histological field in 1.96 mm of total area (AT) and determined by the formula QA = Σ mast cells/AT. The results were expressed as the number of mast cells/mm^2^ (Mandarim‐de‐Lacerda 2003; Leclere et al. 2006).

### 2.6 Oxidative/Nitrosative Stress Markers in the Liver

Frozen liver fragments (100 mg; *n* = 6/group) were homogenized in 1 mL of concentrated phosphate solution (PBS, pH 7.4) and centrifuged at 10,000 × g (12,000 rpm) for 10 min at 4°C. The supernatant was used to determine the activity of antioxidant enzymes, such as superoxide dismutase (SOD), catalase (CAT), and glutathione S‐transferase (GST) (Souza et al. 2019). Results were normalized to nonhomogenized protein levels, and enzyme activity was expressed as units per milligram of protein. A unit of SOD is defined as the amount that inhibits the automatic oxidation rate of pyrogallol by 50%. A unit of CAT activity was defined as the amount of enzyme that decomposes 1 mmol of hydrogen peroxide (H_2_O_2_) during 1 min. A unit of GST activity was defined as the amount of enzyme that catalyzed the formation of 1 μmol of product × min^−1^ × mL^−1^. In addition, lipid peroxidation status was determined by examining tissue levels of malondialdehyde (MDA); supernatant (200 μL) was incubated with thiobarbituric acid to measure the levels of thiobarbituric acid reactive substances. MDA levels were expressed in μmol per milligram of protein. We also measured the total antioxidant capacity (FRAP) according to Benzie and Strain (1996). The FRAP assay consists of a colorimetric measurement of the reduction of the ferric‐tripyridyltriazine complex (Fe^3+^‐TPTZ) to ferrous tripyridyltriazine (Fe^2+^‐TPTZ) by the antioxidants in the sample. The occurrence was performed by adding 10 μL of homogenate to 220 μL of FRAP solution in the microplates. The mixture was incubated for 30 min in the dark and then probed using an ELISA microplate reader. Nitric oxide (NO) levels were indirectly measured in the supernatant by quantifying liver nitrite/nitrate levels according to the Griess methodology (Tsikas 2007). Results were expressed in μmol L^−1^. Finally, protein oxidation was determined by quantifying protein carbonyl (PC) in the pellet (Souza et al. 2019). Results were expressed in nmol mL^−1^, based on the molar extinction coefficient of ε370 = 22 mmol × L^−1^ × cm^−1^. These analyses were performed using an ELISA microplate reader (Multiskan GO, Thermo Scientific). The total protein level in liver tissue was measured using the Bradford method (Bradford 1976).

### 2.7 Trace Element Proportion in the Liver

The trace element content in the liver tissue was estimated using energy dispersive x‐ray spectroscopy coupled with a scanning electron microscope (Leo 1430VP, Carl Zeiss, Jena, Thuringia, Germany) with a silicon drift detector with x‐ray radiation. Frozen samples were dried in an oven at 60°C for 96 h until they reached a constant dry weight. They were then coated with evaporated carbon (Quorum Q150 T, East Grinstead, West Sussex, United Kingdom). The analysis was carried out at 150× magnification using an acceleration voltage of 20 kV and a working distance of 10 nm. The proportion of arsenic, nickel, magnesium, potassium, calcium, manganese, iron, copper, zinc, and selenium was expressed as a percentage.

### 2.8 Activities of Total ATPases, Ca^2+^, Mg^2+^, and Na^+^/K^+^ ATPase

Liver tissue (100 mg; *n* = 6/group) was homogenized in 1 mL of Tris–HCl buffer (0.1 M, pH 7.4) and centrifuged at 1500 × g for 10 min at 4°C. The supernatant was used for the determination of the total (Evans 1969), Ca^2+^ (Hjertén and Pan 1983), Na^+^/K^+^ (Bonting et al. 1962), and Mg^2+^ (Ohnishi et al. 1982) ATPase activity. The ATP solution (0.01 M) was used as a substrate to generate free phosphate by ATPase activity. The reaction was arrested by adding 500 μL of 10% TCA. The tubes were centrifuged at 1500 × g for 10 min. The supernatant was used to measure the phosphorus content using a biochemical kit (Bioclin Laboratories, Belo Horizonte, MG, Brazil) following the manufacturer's instructions. The results were expressed as micrograms (μg) of phosphorus liberated per min per milligram of protein.

### 2.9 Statistical Analysis

The normality of the data was evaluated by the Shapiro–Wilk test. Then, they were submitted to the one‐way analysis of variance test (*one‐way* ANOVA), and means were compared by the post hoc Tukey's test. Differences were significant when *p* < 0.05. Statistical analysis and graphics were performed using GraphPad Prism 7.0 (GraphPad Software Inc., San Diego, CA, USA). Results were expressed as mean ± standard deviation of the mean (SD).

## 3 Results

### 3.1 Biometry and Biochemical Analysis

Results of rats' body weight and liver biometry, including absolute weight, volume, and water content, did not differ between groups (*p* > 0.05; Table 1). In contrast, the liver somatic index was lower in nickel‐exposed animals than in control rats (*p* < 0.05; Table 1). We did not observe any clinical alterations in the animals from all groups during the experiment. Serum ALT, AST, and ALP levels showed no alteration between experimental groups (*p* > 0.05; Table 1).

**TABLE 1:** Metal proportion, biometric parameters, and serum enzyme levels in Wistar rats exposed to arsenic and nickel in drinking water for 70 days.

Parameters	Control	1 mg L^−1^ As	7 mg L^−1^ Ni	1 mg L^−1^ As + 7 mg L^−1^ Ni
Body weight (g)	399.34 ± 9.49^a^	436.31 ± 7.48^a^	438.20 ± 12.81^a^	436.80 ± 16.76^a^
Liver weight (g)	15.22 ± 0.45^a^	15.90 ± 0.67^a^	14.15 ± 0.46^a^	16.09 ± 0.86^a^
Liver somatic index (%)	3.81 ± 0.09^a^	3.65 ± 0.16^ab^	3.24 ± 0.12^b^	3.68 ± 0.13^ab^
Liver volume (mL)	44.00 ± 0.53^a^	45.43 ± 0.71^a^	44.00 ± 0.87^a^	45.43 ± 0.84^a^
Water content (mL/g)	0.69 ± 0.01^a^	0.69 ± 0.01^a^	0.69 ± 0.01^a^	0.68 ± 0.00^a^
ALT (U/L)	49.00 ± 3.72^a^	49.60 ± 2.94^a^	51.80 ± 3.33^a^	47.00 ± 1.38^a^
AST (U/L)	70.03 ± 2.67^a^	86.00 ± 10.23^a^	89.80 ± 8.85^a^	82.00 ± 2.81^a^
ALP (U/L)	174.01 ± 23.27^a^	231.01 ± 19.67^a^	209.80 ± 19.74^a^	171.40 ± 11.46^a^

*Note:* Mean ± SD.

^a,b^Different letters in the same row indicate differences among the groups (*p* < 0.05) by Tukey's test (*n* = 6 animals/group).

### 3.2 Liver Histology

The liver of control rats exhibited a regular parenchymal architecture, with cords of hepatocytes arranged radially in the hepatic lobe, interspersed by sinusoidal capillaries (Figure 1A–C). They also exhibited discrete occurrences of vascular congestion and inflammatory infiltrate (Figure 1A–C). Animals exposed to arsenic presented discrete hemorrhage, moderate vascular congestion, and inflammatory infiltrate, and severe occurrence of hydropic degeneration (Figure 1D–F). Nickel‐exposed animals, in turn, showed discrete hemorrhage and moderate vascular congestion, inflammatory infiltrate, and hydropic degeneration (Figure 1G–I). Finally, rats simultaneously exposed to both chemicals presented discrete hemorrhage and severe vascular congestion, inflammatory infiltrate, and hydropic degeneration (Figure 1J–L).

**FIGURE 1:**
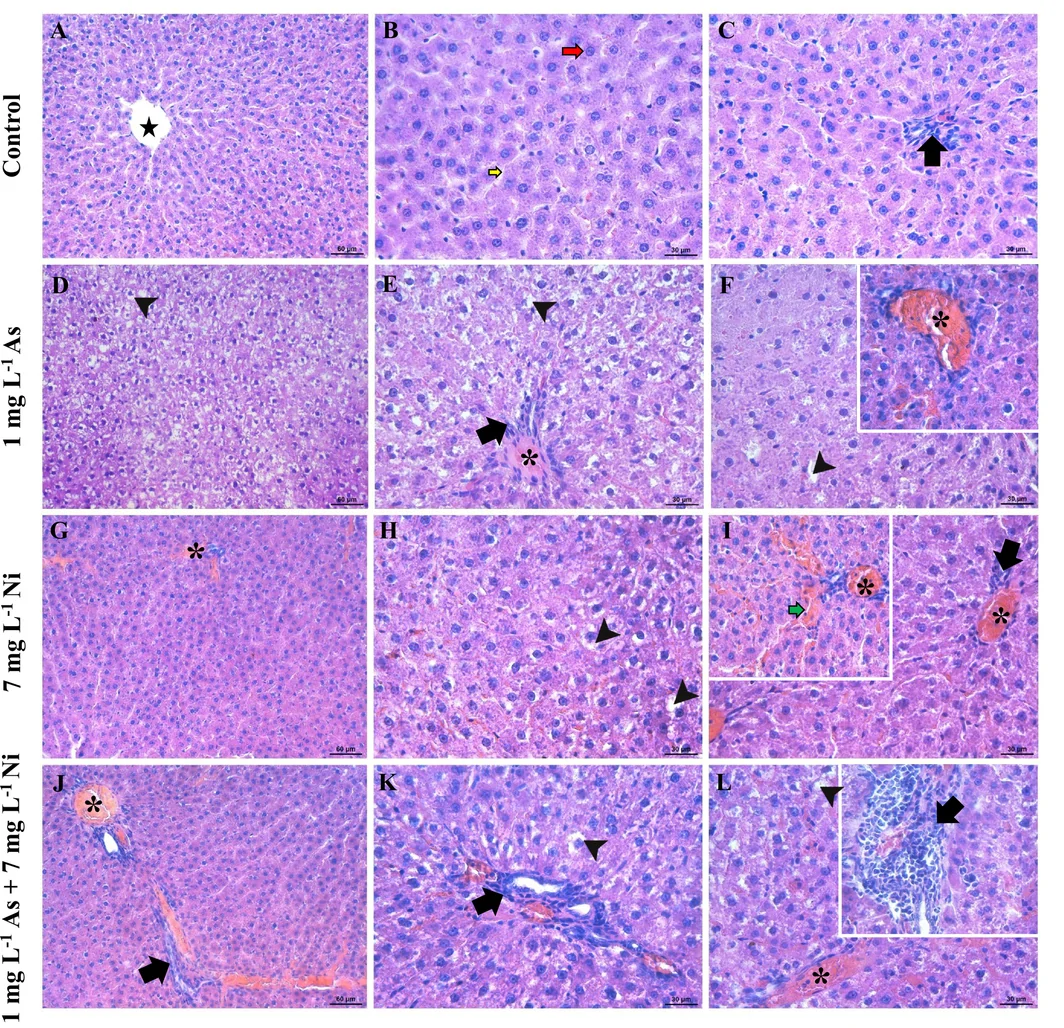
Histological images of the liver from Wistar rats exposed to arsenic (As) and nickel (Ni) in drinking water for 70 days. Figures A, B, and C depict the intact liver parenchyma in control rats, with hepatocytes (red arrow) displayed in cords, interspersed by sinusoidal capillaries (yellow arrow) and blood vessels with an unobstructed lumen (star). Figures D, E, and F show the occurrence of vascular congestion (asterisk), inflammatory infiltrate (black arrow), and hydropic degeneration in the cytoplasm of hepatocytes (arrowhead) from arsenic‐exposed rats. Figures G, H, and I present hepatic alterations, such as vascular congestion, hemorrhage (green arrow), inflammatory infiltrate, and hydropic degeneration, in nickel‐exposed animals. Figures J, K, and L show the presence of vascular congestion, hemorrhage, inflammatory infiltrate, and hydropic degeneration in the liver of co‐exposed rats. Hematoxylin and eosin. Scale bars = 60 μm (A, D, G, J) and 30 μm (B, C, E, F, H, I, K, L).

Stereological analysis of hepatocytes indicated a lower proportion of their nuclei in arsenic‐exposed rats than in the other animals (*p* < 0.05; Table 2). The cytoplasm proportion was lower in rats exposed to arsenic and nickel than in animals from the other groups (*p* < 0.05; Table 2). The proportion of hepatocytes decreased in rats from the three metal‐exposed groups, with the lowest percentage observed in rats receiving As+Ni solutions (*p* < 0.05; Table 2). The liver of co‐exposed rats presented a higher percentage of blood vessels and sinusoid capillaries than the liver of control animals (*p* < 0.05; Table 2). Macrophage percentage did not change between the experimental groups (*p* > 0.05; Table 2).

**TABLE 2:** Proportion of liver parenchyma components in adult Wistar rats exposed to arsenic and nickel in drinking water for 70 days.

Parameters (%)	Control	1 mg L^−1^ As	7 mg L^−1^ Ni	1 mg L^−1^ As + 7 mg L^−1^ Ni
Nucleus hepatocyte	8.73 ± 0.37^a^	5.33 ± 0.19^b^	8.21 ± 0.54^a^	7.55 ± 0.46^a^
Cytoplasm hepatocyte	63.65 ± 0.62^a^	60.76 ± 1.51^a^	58.43 ± 1.55^a^	52.18 ± 1.57^b^
Hepatocyte	72.38 ± 0.31^a^	65.82 ± 1.50^b^	66.55 ± 1.07^b^	59.73 ± 1.76^c^
Sinusoid capillaries	25.11 ± 0.41^a^	26.65 ± 0.61^ab^	27.31 ± 0.72^ab^	29.54 ± 1.10^b^
Macrophages	1.03 ± 0.11^a^	1.06 ± 0.13^a^	1.35 ± 0.09^a^	1.63 ± 0.09^a^
Blood vessels	1.37 ± 0.53^a^	6.15 ± 0.80^ab^	3.91 ± 1.44^ab^	9.05 ± 2.66^b^

*Note:* Mean ± SD.

^a,b,c^Different letters in the same row indicate differences among the groups (*p* < 0.05) by Tukey's test (*n* = 6 animals/group).

The percentage of hepatocyte glycogen increased in rats receiving As+Ni solutions compared to animals from the other groups (*p* < 0.05; Figure 2A–E). Moreover, the percentage of collagenous fibers stained with Masson's Trichrome (Figure 2F–I) increased in the liver of co‐exposed rats compared to control and nickel‐exposed rats (*p* < 0.05; Figure 2J), while the percentage of fluorescent type 1 collagen in the Sirius red stain (Figure 2K–N) increased in co‐exposed animals compared to their controls (*p* < 0.05; Figure 2O). Moreover, the number of mast cells was higher in the liver of rats from the Ni and As+Ni groups than in the liver of control animals (*p* < 0.05; Figure 2P–T).

**FIGURE 2:**
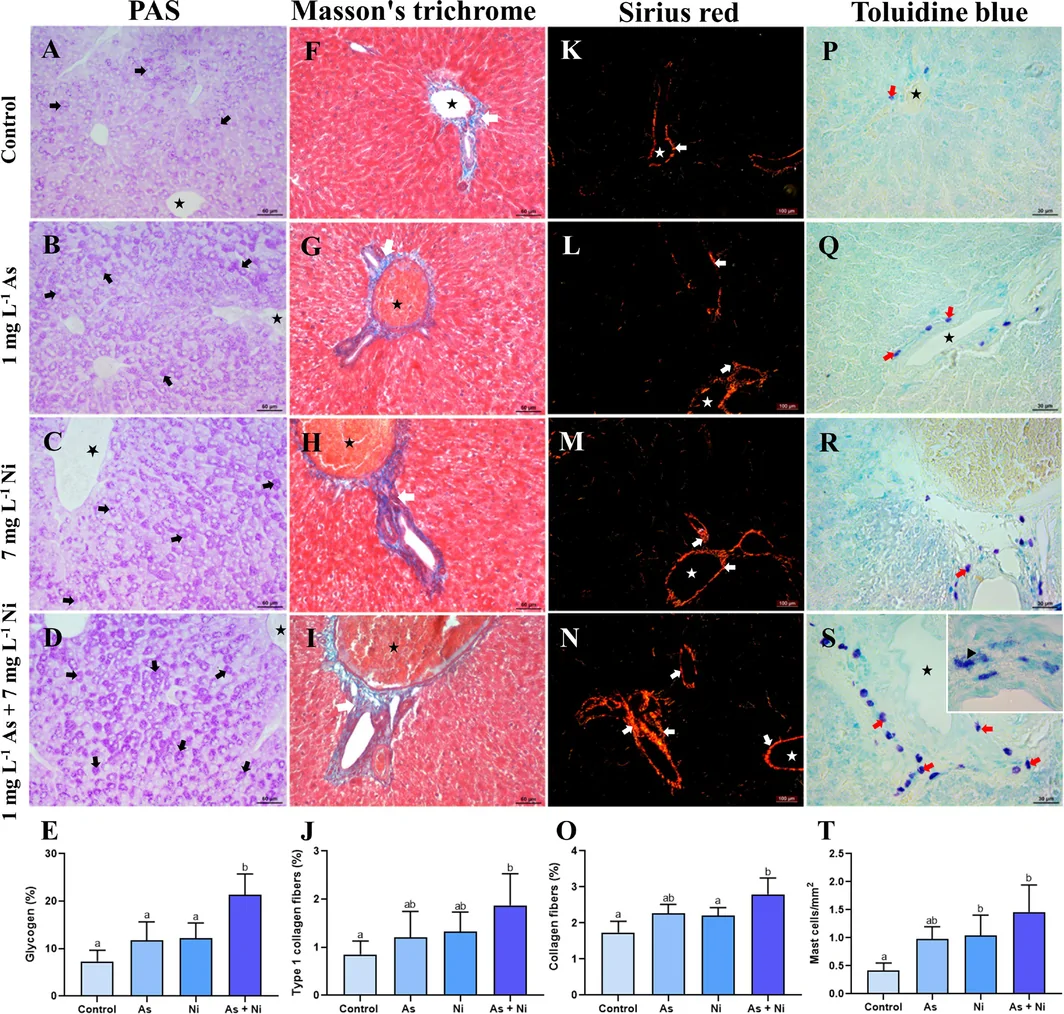
Photomicrographs of the liver from Wistar rats exposed to arsenic (As) and nickel (Ni) in drinking water for 70 days. Figures A, B, C, and D show the presence of glycogen aggregates (black arrows) in the liver tissue stained with periodic acid–Schiff (scale bar: 60 μm). Figures F, G, H, and I present collagen stained in blue by Masson's Trichrome stain (Scale bar: 60 μm). Figures K, L, M, and N show the presence of collagen (white arrows) in the liver stained with Sirius red (Scale bar: 100 μm). Figures P, Q, R, and S show the presence of mast cells (red arrows and black arrowheads) in the hepatic tissue (toluidine blue; scale bar: 30 μm). Star: blood vessels. Bar graphs represent the proportion of glycogen (E), type 1 collagen fibers (J), total collagen fibers (O), and mast cell count (T). Mean ± SD. ^a,b^Different letters on the same row indicate differences among the groups (*p* < 0.05) by Tukey's test. (*n* = 6 animals/group).

### 3.3 Oxidative/Nitrosative Stress Markers in the Liver

The hepatic SOD and GST activity did not differ between groups (*p* > 0.05; Figure 3A,C). The activity of CAT was lower in co‐exposed rats than in their controls (*p* < 0.05; Figure 3B). In contrast, MDA and FRAP levels did not differ between experimental groups (*p* > 0.05; Figure 3D,E). NO production was higher in the liver of animals exposed to arsenic and nickel than in control rats (*p* < 0.05; Figure 3F). Moreover, PC production was higher in the liver of nickel‐exposed rats than in the control (*p* < 0.05; Figure 3G).

**FIGURE 3:**
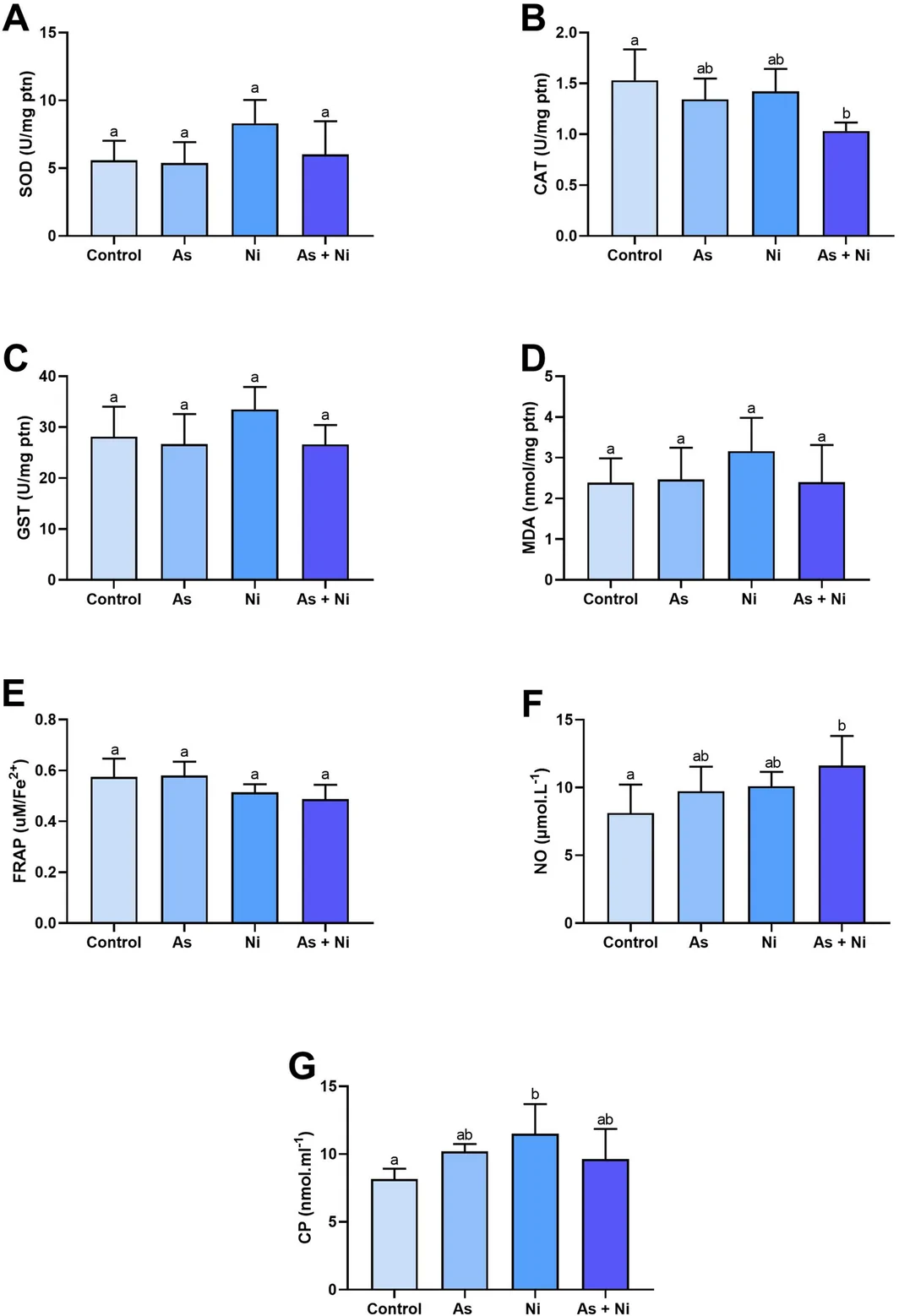
Antioxidant enzyme activity and oxidative/nitrosative markers in the liver of rats exposed to arsenic (As; 1 mg L^−1^), nickel (Ni; 7 mg L^−1^), and As+Ni (1 mg L^−1^ and 7 mg L^−1^, respectively) for 70 days. SOD = superoxide dismutase (A); CAT = catalase (B); GST = glutathione S‐transferase (C); MDA = malondialdehyde (D); FRAP = total antioxidant capacity (E); NO = nitric oxide (F); PC = protein carbonyl (G). Mean ± SD. ^a,b,c^Different letters on the same row indicate differences among the groups (*p* < 0.05) by Tukey's test (*n* = 6 animals/group).

### 3.4 Proportion of Trace Elements in the Liver

Livers of rats exposed to arsenic and nickel individually, as well as co‐exposed rats, presented a higher proportion of these elements than their controls (*p* < 0.05; Table 3). Regarding other trace elements, co‐exposed rats had a lower percentage of iron than their controls, whereas nickel‐exposed animals exhibited higher percentages of calcium and copper than rats from the control group (*p* < 0.05; Table 3). The proportion of magnesium, potassium, manganese, zinc, and selenium was not altered between groups (*p* > 0.05; Table 3).

**TABLE 3:** Proportion of microelements in the liver of adult Wistar rats exposed to arsenic and nickel in drinking water for 70 days.

Parameters	Control	1 mg L^−1^ As	7 mg L^−1^ Ni	1 mg L^−1^ As + 7 mg L^−1^ Ni
Arsenic	1.35 ± 0.64^a^	6.21 ± 1.16^b^	1.29 ± 1.80^a^	5.27 ± 1.85^b^
Nickel	1.36 ± 0.94^a^	1.28 ± 0.96^a^	6.37 ± 2.99^b^	6.18 ± 1.99^b^
Magnesium	26.39 ± 3.74^a^	28.19 ± 4.73^a^	22.25 ± 9.06^a^	25.58 ± 5.58^a^
Potassium	6.29 ± 3.9^a^	5.66 ± 2.03^a^	6.95 ± 3.81^a^	7.39 ± 3.35^a^
Calcium	2.39 ± 0.89^a^	2.94 ± 1.96^ab^	5.47 ± 1.19^b^	1.97 ± 2.58^ab^
Manganese	3.79 ± 1.18^a^	3.73 ± 0.69^a^	4.98 ± 2.39^a^	1.28 ± 0.99^a^
Iron	3.87 ± 0.94^a^	4.26 ± 2.59^ab^	1.19 ± 2.39^ab^	1.6 ± 0.48^b^
Copper	12.71 ± 1.37^a^	13.97 ± 3.42^ab^	21.09 ± 3.59^b^	9.08 ± 6.34^ab^
Zinc	12.43 ± 2.29^a^	8.02 ± 2.71^a^	6.10 ± 3.91^a^	11.38 ± 2.35^a^
Selenium	28.70 ± 4.47^a^	25.78 ± 4.27^a^	24.36 ± 5.32^a^	30.40 ± 4.70^a^

*Note:* Mean ± SD.

^a,b^Different letters in the same row indicate differences among the groups (*p* < 0.05) by Tukey's test (*n* = 6 animals/group).

### 3.5 Activity of Total, Ca^2+^, Mg^2+^, and Na^+^/K^+^ ATPases

Rats receiving nickel alone and combined with arsenic showed lower total ATPase activity than control animals (*p* < 0.05; Table 4). Animals exposed only to nickel presented higher Ca^2+^ ATPase pump activity in the liver than rats exposed to arsenic and both metals (*p* < 0.05; Table 4). The Na^+^/K^+^ and Mg^2+^ ATPase activities did not change between the experimental groups (*p* > 0.05; Table 4).

**TABLE 4:** Activity of total ATPase, Ca^2+^, Na^+^/K^+^, and Mg^2+^ ATPases in the liver of adult Wistar rats exposed to arsenic and nickel in drinking water for 70 days.

Parameters (Pi/min/mg/protein)	Control	1 mg L^−1^ As	7 mg L^−1^ Ni	1 mg L^−1^ As + 7 mg L^−1^ Ni
Total ATPase activity	0.026 ± 0.001^a^	0.024 ± 0.001^ab^	0.023 ± 0.0002^b^	0.022 ± 0.001^b^
Ca^2+^ ATPase activity	0.008 ± 0.0001^ab^	0.008 ± 0.0003^a^	0.009 ± 0.0002^b^	0.007 ± 0.0002^a^
Na^+^/K^+^ ATPase activity	0.011 ± 0.0005^a^	0.012 ± 0.0006^a^	0.012 ± 0.0005^a^	0.011 ± 0.0003^a^
Mg^2+^ ATPase activity	0.010 ± 0.0002^a^	0.009 ± 0.0006^a^	0.010 ± 0.0005^a^	0.009 ± 0.0002^a^

*Note:* Mean ± SD.

^a,b^Different letters in the same row indicate differences among the groups (*p* < 0.05) by Tukey's test (*n* = 6 animals/group).

## 4 Discussion

Our findings provide pioneering insights into the effects of subchronic exposure to arsenic and nickel on liver morphology and function. This experiment is relevant to understanding the mechanisms of metal toxicity, as humans are exposed to mixtures of elements rather than a single element (Sani et al. 2023). Under our experimental conditions, 7 mg L^−1^ of nickel but not 1 mg L^−1^ of arsenic triggered ionic and oxidative imbalance, as well as mitochondrial ATPase dysfunction. Inversely, none of the exposed rats showed alterations in hepatic MDA levels or in serum ALT, AST, and ALP, indicating intact hepatocyte membrane integrity. Nonetheless, rats exposed to arsenic and nickel, alone or in combination, presented histological alterations whose intensity varied across experimental groups. Our findings confirmed the hypothesis that co‐exposure to arsenic and nickel intensified liver damage in rats. We summarized the primary outcomes in Figure 4, demonstrating the cause‐and‐effect of the arsenic–nickel interaction.

**FIGURE 4:**
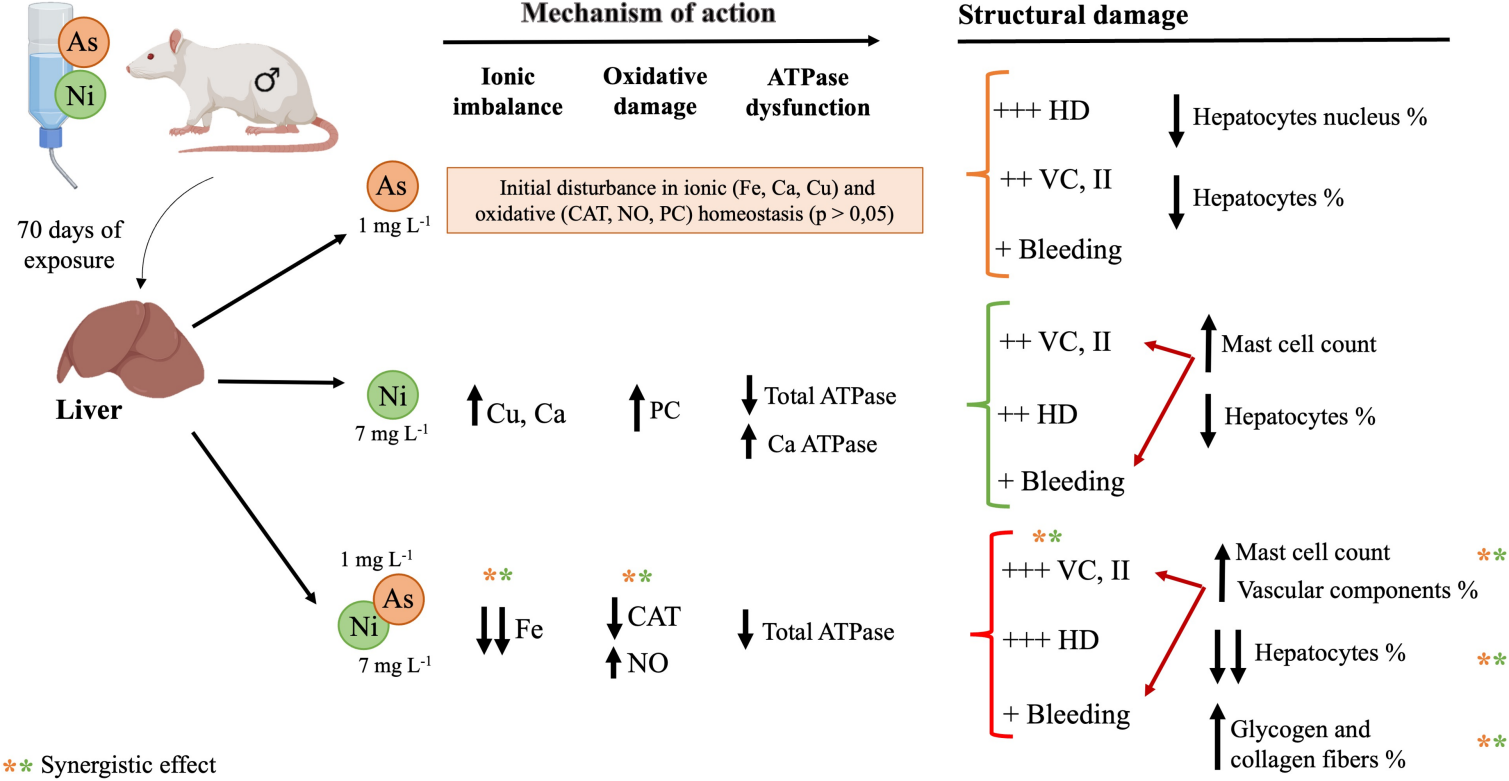
Schematic drawing depicting the mechanism of metal toxicity and the primary outcomes from the rat liver after subchronic exposure to arsenic (As) and nickel (Ni), alone and combined. Arsenic at 1 mg L^−1^ triggered an initial disruption in ionic and oxidative homeostasis, but it was insufficient to alter the hepatic microenvironment significantly. On the other hand, nickel at 7 mg L^−1^ broke down ionic homeostasis (high proportion of calcium [Ca] and copper [Cu]), promoted protein oxidation (PC), and disturbed total and Ca^2+^ ATPases activity. The combined exposure to both chemicals acted synergistically by decreasing the iron (Fe) proportion and catalase (CAT) activity, increasing nitric oxide (NO) production, and reducing total ATPase activity. Histological findings revealed the occurrence of hydropic degeneration (HD), vascular congestion (VC), inflammatory infiltrate (II), and bleeding at discrete (+), moderate (++), and severe (+++) intensity. Finally, quantitative analysis showed alteration in the volumetry of hepatic components (nucleous, hepatocyte, glycogen), vascular components (blood vessels and sinusoid capillaries), collagen fibers, and mast cell count.

The results showed retention of arsenic and nickel in the hepatic tissue of exposed and co‐exposed rats. Arsenite enters the hepatocyte via aquaglyceroporins 9 and glucose transporters (GLUT), whereas nickel chloride uses calcium or iron channels and divalent cation transporters (DMT‐1) to reach the cytosol (Arnich et al. 2000; Davidson et al. 2005; Garbinski et al. 2019). Nickel is first excreted in the urine, and its remnant content can then accumulate in the liver and other organs (Arnich et al. 2000), unlike arsenic, which reaches the liver after its intestinal absorption (Machado‐Neves and Souza 2023). The strong affinity for sulfhydryl (thiol) groups may retain arsenic and nickel in body tissues, such as the liver, compromising the function of thiol‐containing proteins. About 27% of the nickel and 70%–90% of the arsenic ingested through drinking water are taken up by cells (Tokar et al. 2012). Therefore, the concentration of metals administered to animals should reflect the rate of metal absorption in toxicological studies using metal mixtures. For instance, Owumi et al. (2020) and Sharma et al. (2024) used nickel concentrations higher than those of cadmium in their experiments, as we did here with nickel and arsenic.

Within the cell, nickel and arsenic can break down ionic and oxidative homeostasis (Machado‐Neves 2022; Rahimzadeh et al. 2025). However, our results showed that nickel was the only metal to elicit an increase in calcium and copper content after isolated exposure (Figure 4). Nickel can interact with calcium and copper due to their similar divalent nature. It may increase intracellular calcium by inhibiting calcium channels, activating calcium‐sensing receptors, and releasing calcium from internal stores (Utsunomiya et al. 2021). In the case of copper, its relationship with nickel seems to be antagonistic, relying on the animal's nutritional condition and iron presence (Nielsen et al. 1984). Despite the statistics indicating a nonsignificant effect of nickel on iron content (low proportion), as well as arsenic (high iron proportion) after isolated exposure, the combination of these metals significantly reduced iron in the rat liver (Figure 4). Indeed, nickel may disturb iron metabolism by competing for DMT‐1 (Angelova et al. 2014), affecting its transport into the cell and reducing its levels, as observed here. In addition, nickel may compete for iron sites on enzymes (Ilina et al. 2020), such as the prolyl hydroxylases that, consequently, modify hypoxia inducible factor 1 alpha (HIF‐1α; Chen et al. 2005). This disturbance dysregulates the cell's ability to exert diverse functions and creates an intracellular state of hypoxia due to the stabilization and transactivation of HIF‐1α, even under normal oxygen tension (Davidson et al. 2005; Rahimzadeh et al. 2025). Arsenic, in turn, competes with iron‐enzyme binding sites, increasing free iron levels that may trigger oxidative stress via the Fenton reaction (Winterbourn 1995; Machado‐Neves and Souza 2023).

Additionally, rats exposed to nickel alone showed high levels of carbonylated proteins in the hepatic tissue. Metal‐catalyzed protein oxidation is one of the most common mechanisms for inducing protein oxidation, introducing carbonyl groups in the presence of H_2_O_2_, iron, and copper. The main product of protein oxidation is PC, which favors the formation of protein aggregates and the loss of enzymatic activity (Dalle‐Donne et al. 2003; Cecarini et al. 2007). Precisely, reactive species can oxidize proteins, damaging several sites on their side chains and backbones. The variety of reaction sites generates a wide range of posttranslational protein modifications, consequently modifying the composition and folding, net charge, and hydrophobicity/hydrophilicity of proteins. These alterations may affect the functionality of receptors, enzymes, carriers, or structural proteins (Kehm et al. 2021).

The co‐exposure, in turn, intensified the oxidative imbalance by lowering CAT activity and increasing NO production. In the case of CAT, we might infer that SOD could convert superoxide anions into H_2_O_2_, thereby raising tissue H_2_O_2_ levels. High H_2_O_2_ levels may have forced more CAT activity until exhaustion, once this enzyme converts H_2_O_2_ into water and O_2_ to protect cells against oxidative damage (Bisht et al. 2017). CAT activity may also be affected by the low iron proportion in the liver of co‐exposed animals, since this metal is an essential cofactor of CAT.

Interestingly, arsenic at 1 mg L^−1^ did not elicit a significant ionic and oxidative imbalance after exposure alone. Despite a nonsignificant result, this metalloid triggered an initial disturbance in ionic balance (high iron, copper, and calcium content) and antioxidant enzyme activity (low CAT), accompanied by high NO and PC levels. This initial injury may have contributed to driving oxidative alterations in the liver of co‐exposed rats. Our group has reported that 1 mg L^−1^ of arsenic altered only SOD activity in the prostate (Coimbra et al. 2024) and observed a disruption of antioxidant enzyme activity in the testis (low CAT and high GST activity) of arsenic‐exposed rats at the same concentration (unpublished data), without producing high levels of oxidative byproducts. It is noteworthy that the liver plays a crucial role in metabolic and detoxification processes. For that reason, it has a robust antioxidant defense system consisting of enzymatic and nonenzymatic components. Besides the antioxidants traditionally assessed in toxicological studies, such as SOD, CAT, GST, and FRAP (Adedara et al. 2014; Souza et al. 2018), enzymes from the glutathione family (e.g., glutathione peroxidase and reductase), peroxiredoxins, and thioredoxins are important components of redox defense (Allameh et al. 2023). Herein, the antioxidant machinery might be struggling to sustain ROS at controlled levels, protecting hepatic tissue against oxidative damage. The result of MDA levels may confirm it, along with the serum enzymatic profile, suggesting the maintenance of hepatocyte membrane integrity (Reagan et al. 2013) under our experimental conditions.

Nonetheless, co‐exposed rats exhibited elevated hepatic NO levels. NO is a versatile signaling molecule that acts as a double‐edged sword in oxidative stress and inflammation. Endothelial cells lining blood vessels synthesize NO by deaminating L‐arginine to L‐citrulline via NO synthase, thereby regulating blood flow. In addition, NO is a potent vasodilator that relaxes and widens blood vessels and acts as an anti‐inflammatory agent. At high levels, however, NO may promote inflammation and tissue injury, making it a relevant marker of nitrosative stress (Pierini and Bryan 2015). NO can interact with superoxide anion to form peroxynitrite, a highly reactive nitrogen species that causes severe lipid, protein, and DNA damage (Cecarini et al. 2007). Herein, NO appears to be more closely associated with vascular and inflammatory processes. For instance, the liver of co‐exposed rats exhibits an increased proportion of vascular components (blood vessels and sinusoidal capillaries; Figure 4) and a severe occurrence of vasodilatation and inflammatory infiltrate, which occurred at moderate intensity in rats after individual metal exposure. A high proportion of vascular components may have contributed to the low proportion of cytoplasm and hepatocytes observed in all exposed animals, particularly in co‐exposed rats.

Furthermore, rats exposed to nickel, alone or in combination with arsenic, showed a high number of mast cells in liver tissue (Figure 4). Mast cells are allergy‐mediated cells that can regulate histaminergic responses when activated (Huang et al. 2022). We know that NO exerts regulatory activity on these cells (Coleman 2002), stimulating the release of histamine and pro‐inflammatory cytokines. These substances mediate downstream signaling pathways that culminate in increased vessel permeability and local leukocyte recruitment (Swindle and Metcalfe 2007; Huang et al. 2022). Notwithstanding, there are differences in the way arsenic and nickel affect mast cell recruitment and degranulation. The metalloid disrupts mast cell degranulation by interfering with early signaling steps, such as tyrosine phosphorylation and calcium influx (Shim et al. 2016), thereby preventing its recruitment and activation. Conversely, nickel acts as a potent stimulator of mast cell‐mediated inflammation via the TLR4 pathway, as described in skin inflammation (Saito et al. 2016).

The activity of mitochondrial ATPases was also affected by nickel, alone or in combination with arsenic. Specifically, the total ATPase activity was lower in rats from the nickel and co‐exposed groups, whereas Ca^2+^ activity increased in nickel‐exposed animals (Figure 4). The disruption of calcium and iron homeostasis caused by heavy metals might have influenced these results. Unlikely, the potassium and magnesium content did not change in the liver of metal‐exposed rats, which may have influenced the maintenance of Na^+^/K^+^‐ATPase and Mg^2+^‐ATPase activity. Membrane‐bound ATPases play roles in osmotic balance, ionic regulation, and membrane permeability, thereby sustaining osmoregulation of ion concentrations and extracellular fluids (Dogan et al. 2015; Atli et al. 2019). Thus, the lack of ATPase activity may have disrupted osmoregulation and disturbed water and electrolyte homeostasis, leading to the hydropic degeneration observed in hepatocytes across all exposed groups. This pathology may involve a dysfunction in water distribution between the intra‐ and extracellular compartments (Susilo et al. 2019), without edema. The absence of edema, as well as the absence of alterations in the biometric parameters assessed here, may support this statement. The lower LSI index observed in nickel‐exposed animals was due to their high body weight, with no influence of metal exposure.

Other microscopic alterations that occurred in the liver of co‐exposed rats were the high proportion of glycogen and collagen fibers in hepatic cells and stroma, respectively (Figure 4). The intracellular accumulation of hepatic glycogen may be a sign of hepatotoxicity or a disorder in carbohydrate metabolism (Rogers et al. 2002). Arsenic may disturb glucose metabolism mainly in its pentavalent form, but arsenite may trigger enzymatic disorders due to its affinity to thiol groups (Machado‐Neves and Souza 2023). Nickel may induce glycogen accumulation in hepatocytes by disrupting glucose and insulin regulation through reducing GLUT‐2 and glucokinase genes critical for glucose uptake and insulin secretion (Feezan et al. 2024). Moreover, the accumulation of collagen bordering vessels and capillaries may be an essential feature of the hepatic response to infectious, toxic, or metabolic injury (Friedman 1990). Metal retention can induce an initial collagen deposition, even if there is no increase in the organ weight. The increase in the percentage of connective tissue surrounding veins, preceded by an apoptotic process, may be related to fibrosis with deposition of type I collagen (Rauterberg et al. 1981). The increase in collagen fibers can hinder vascular remodeling, resulting in communication problems between parenchymal components and damage to the organ's function (Mouro et al. 2019).

## 5 Conclusions

Our findings support the hypothesis that co‐exposure to arsenic and nickel intensifies hepatic tissue damage in Wistar rats after subchronic exposure. Together, arsenic and nickel caused a reduction in iron proportion and CAT activity, an increase in NO levels, mast cell count, vascular components volumetry, and intensified the severity of vascular congestion and inflammatory infiltration in the liver tissue. The mechanism underlying arsenic–nickel interaction included ionic imbalance, oxidative damage, and ATPase dysfunction. Nickel at 7 mg L^−1^ elicited most of the alterations observed after isolated exposure, disturbing copper and calcium proportion, oxidizing proteins, altering Ca^2+^ and total ATPases, and recruiting mast cells. Arsenic at 1 mg L^−1^, in turn, initiated ionic and oxidative disturbances that, during an individual exposure for 70 days, did not affect those parameters significantly. Collectively, isolated and combined exposure to arsenic and nickel may intensify the damage observed here whether in a prolonged exposure period. This study provides evidence that metal mixtures induce significant hepatic injury through mechanisms distinct from isolated exposures, highlighting the importance of considering chemical interactions in metal mixtures, as their combined effects can trigger distinct toxicological pathways that result in structural damage.

## Author Contributions

**Thainá Iasbik‐Lima:** conceptualization, formal analysis, investigation, methodology, writing – original draft. **Luiz Otávio Guimarães‐Ervilha:** formal analysis, investigation, writing – original draft. **Isabela Pereira da Silva Bento:** investigation. **Renê Chagas da Silva:** methodology, resources. **Mariana Machado‐Neves:** conceptualization, funding acquisition, project administration, supervision, writing – review and editing.

## Funding

This work was supported by the Coordenação de Aperfeiçoamento de Pessoal de Nível Superior (CAPES, Grant Number 88887.584671/2020‐00), Fundação de Amparo à Pesquisa do Estado de Minas Gerais (FAPEMIG, Grant Number PPM‐00621‐18, APQ‐00361‐23; BPD‐00843‐22 to M.M.‐N.) and Conselho Nacional de Desenvolvimento Científico e Tecnológico (CNPq, Grant Number 313524/2021‐1).

## Conflicts of Interest

The authors declare no conflicts of interest.
